# Perceptions on the Economic Feasibility of Sustainable Roundworm Control Practices in Grazed Livestock—A Short Survey Among European Farmers and Veterinarians

**DOI:** 10.3390/ani16101552

**Published:** 2026-05-19

**Authors:** Hannah Njiriku Mwangi, Leen Lietaer, Edwin Claerebout, Laura Rinaldi, Antonio Bosco, Smaragda Sotiraki, Marcin Mickiewicz, Mahmut Sinan Erez, Esma Kozan, Annick Spaans, Carole Toczé, Natascha Meunier, Maria Martínez Valladares, Jarosław Kaba, Mickael Bernard, Adrian-Valentin Potârniche, Aija Malniece, Tomas Kupčinskas, Dave Bartley, Johannes Charlier, Tong Wang

**Affiliations:** 1 Kreavet, Hendrik Mertensstraat 17, 9150 Kruibeke, Belgium; hannah@kreavet.com (H.N.M.);; 2 Department of Social Sciences, ILVO, Burgemeester Van Gansberghelaan 92/1, 9820 Merelbeke, Belgium; 3 Laboratory of Parasitology, Faculty of Veterinary Medicine, Ghent University, Saliburylaan 133, 9820 Merelbeke, Belgium; 4 CREMOPAR, Department of Veterinary Medicine and Animal Production, University of Naples Federico II, 80137 Napoli, Italy; 5 Hellenic Agricultural Organisation-Dimitra (ELGO-DIMITRA), 57001 Thessaloniki, Greece; 6 Division of Veterinary Epidemiology and Economics, Institute of Veterinary Medicine, Warsaw University of Life Sciences, 02-776 Warsaw, Poland; 7 Toinen Pro Art Fundacja, 99-440 Zduny, Poland; 8 Faculty of Veterinary Medicine, Afyon Kocatepe University, 03200 Afyonkarahisar, Türkiye; 9 ZLTO—Zuidelijke Land- en Tuinbouworganisatie Vereniging, 5222 Den Bosch, The Netherlands; 10 IDELE—Institut de l’Élevage, 31321 Castanet-Tolosan, France; 11 Animal Health Ireland, N41 WN27 Carrick-on Shannon, County Leitrim, Ireland; 12 Instituto de Ganadería de Montaña, Animal Health Department, CSIC-University of Leon, Grulleros, 24346 León, Spain; 13 CIIRPO—Centre Interregional d’Information et de Recherche en Production Ovinele Mourie, 87260 Saint-Priest-Ligoure, France; 14 Department of Infectious Diseases and Preventive Medicine, University of Agricultural Sciences and Veterinary Medicine, 400372 Cluj-Napoca, Romania; 15 Clinical Institute, Faculty of Veterinary Medicine, Latvia University of Life Sciences and Technologies, 3004 Jelgava, Latvia; 16 Department of Veterinary Pathobiology, Lithuanian University of Health Sciences, 47140 Kaunas, Lithuania; 17 Moredun Research Institute, Penicuik EH26 0PZ, UK

**Keywords:** gastrointestinal nematodes, sustainable worm control, milk yield, weight gain, grazing ruminants

## Abstract

Gastrointestinal nematodes (GIN) continue to impose substantial health and productivity losses in grazing ruminants, and the accelerating emergence of anthelmintic resistance (AR) underscores the need for SWC strategies. Although multiple SWC approaches have been validated experimentally, their implementation across European livestock systems remains inconsistent, and limited evidence exists regarding the stakeholders’ perceptions that affect decision-making. This study conducted a multilingual cross-sectional survey of 1261 respondents, including farmers, veterinarians, advisors, and other professionals, across 13 European countries to evaluate perceived worm-control cost burdens and the economic feasibility of seven SWC strategies. Descriptive and regression analyses revealed that a majority of respondents (56.7%) considered diagnostic testing to be financially reasonable, although perceptions varied significantly between countries. Sustainable anthelmintic use, quarantine and strategic screening, and grazing management were perceived as the most viable strategies, whereas biological control and bioactive compound-based approaches elicited greater uncertainty. An aggregated SWC Attitude Score demonstrated systematically higher acceptance among veterinarians compared to farmers, while male and older respondents exhibited lower levels of agreement across practices. The overall findings suggest that economic considerations may not be perceived as the primary barrier to sustainable worm control adoption, but other practical factors may potentially limit implementation.

## Simple Summary

Sustainable worm control (SWC) is important for addressing the growing problem of roundworm resistance to deworming drugs. However, SWC approaches are often more complex to undertake than current treatment-based practices, and we do not yet know how farmers, veterinarians and other stakeholders perceive the economic viability of such options for managing roundworm infections. In this short study, we asked over a thousand participants from 13 European countries about their perceptions of the economic feasibility of seven selected SWC approaches. Most respondents believe these practices are worth the investment and can deliver economic benefits. However, opinions varied across countries and stakeholder groups, indicating that advice and communication need to be adapted to each region so farmers and veterinarians can implement SWC more easily and practically.

## 1. Introduction

Gastrointestinal nematodes (GIN) remain one of the most significant challenges to the health, welfare, and productivity of grazing ruminants globally. Traditionally, control has relied heavily on the frequent and prophylactic use of anthelmintics; however, the rapid emergence of anthelmintic resistance (AR) in many GIN has rendered this approach increasingly unsustainable [1,2,3,4]. It is crucial for the European livestock sector to undergo a ‘green transition’, seeking sustainable worm control (SWC) approaches that maintain animal performance while minimizing the use of chemical products. However, despite the growing availability of SWC approaches, their adoption tends to remain uneven among farmers. Lack of data on the economic impact of SWC approaches is considered an important barrier to their implementation [5]. Additionally, farmers often abandon long-established worming routines only when both economic analyses and scientific evidence clearly demonstrate the value of alternative strategies, a point emphasized by [6]. Similarly, Howell et al. (2025) identify “time and cost” as practical, real-world barriers hindering the uptake of sustainable GIN control approaches among farmers [7,8].

It is important to understand the factors that influence key stakeholders’ decision-making in considering adoption of SWC practices and whether they consider the practices to be credible, acceptable and actionable. Hence, this paper provides more data on this aspect by conducting a short, wide European survey on the perceptions of economic feasibility. Below, we briefly introduce the different SWC approaches that were evaluated in the survey, followed by the concrete objectives of this paper.

Current research has identified a diverse ‘toolbox’ of promising SWC approaches such as targeted (selective) treatment TST, Targeted Treatment (TT), grazing management, selective breeding, vaccination, biological control, use of diagnostics, nutrition, including bioactive forages [9]. While for most SWC approaches, proof of concept has been delivered that they can effectively reduce worm burdens while reducing anthelmintic use, their implementation in livestock systems remains limited [9]. Among all the options, TT and TST may be the most thoroughly validated, focusing on maintaining parasite “refugia”- populations of worms unexposed to drugs to slow the development of resistance [10,11]. TST involves treating only specific individuals based on diagnostic indicators, whereas TT targets the whole group only when specific risk thresholds are reached [12,13]. TST is known to reduce anthelmintic reliance; recent meta-analyses reported that a statistically significant but small production cost must often be accepted in terms of animal performance [14].

Other complementary sustainable control approaches include grazing and direct feeding of bioactive plants and/or plant-derived compounds such as condensed tannins and saponins provided through plant multispecies [15], bioactives (additives given on top of the normal diet that contain microorganisms, enzymes, or other bioactive compounds [16,17]. Additionally, agro-industrial by-products, such as pomegranate peel, grape pomace, and hazelnut skins, have been found to contain condensed tannins and polyphenols, which have the potential to inhibit GIN larval development and reduce parasite fecundity [18]. However, the effectiveness and host immune responses of bioactive plants vary, as concentrations of active compounds are influenced by climate, processing, and harvest or feeding time [9,15].

Biological control agents such as the nematode-trapping fungus *Duddingtonia flagrans* can reduce the number of infective larvae on pasture [19,20,21]. However, their efficacy depends on environmental factors, including temperature, humidity, and pasture conditions, which influence fungal growth and trapping activity [19]. In terms of other grazing management, specific strategies such as mixed grazing (two or more animal species graze together on the same pasture) have been shown to improve production performance in goats and cattle, yet economic modeling indicates that the financial return varies significantly between farms, requiring site-specific evaluation [22,23,24].

Conducting quarantine and strategic screening involves isolating new or returning stock to prevent the introduction of infectious agents, especially resistant GIN, into the existing herd or flock. This follows a specifically recommended sequence: isolate, test, treat, quarantine turnout, test again, and merge [25,26]. Selective breeding of animals resistant to or resilient to GIN infections offers a clear long-term productivity advantage [27,28]. With very few nematode vaccines available in the field, and only marketed in certain countries, there is a significant lack of data regarding their effects on long-term animal productivity or farm profitability in Europe [29].

A significant gap remains regarding the practical adoption of SWC practices [30]. Ultimately, the feasibility of these methods depends on the perceptions of farmers and veterinarians; understanding their beliefs regarding the reliability and day-to-day manageability of these practices is essential to ensure their long-term implementation. Furthermore, the economic burden of worm control is not uniform across European countries, sectors, and individual farms. Variations in anthelmintic pricing, livestock margins, labor costs, and regional agricultural policies create diverse financial contexts [31]. These factors shape how stakeholders evaluate the affordability and perceived value of SWC approaches, leading to distinct regional perceptions across Europe.

While earlier research has focused mainly on awareness, attitudes, and behavioral barriers related to parasite control, there is limited evidence on how different SWC approaches are perceived in terms of their economic feasibility and practical applicability under real-world livestock conditions. To address this gap, we conducted a large, multilingual, cross-sectional European survey to systematically assess how farmers, veterinarians, and other livestock stakeholders perceive the economic feasibility of SWC approaches. This survey was conducted as part of the Horizon Europe Thematic Network-Sustainable Parasite Control (SPARC).

This study had two main objectives: (i) to assess the level of endorsement of various SWC approaches among key stakeholders, including farmers, veterinarians, and farm advisors; and (ii) to understand the perceived economic burden and feasibility of these practices. Generated insights can support the future focus and further development of SWC approaches across Europe. This perception survey is just the first step within the SPARC project, and the hard financial data (which is being collected from the pilot farms of the same project) will, in the future, be used to validate it.

## 2. Materials and Methods

### 2.1. Survey Design

A survey was administered electronically between February and June 2025 to capture information on perceptions and opinions of veterinarians, farmers and other stakeholders of ruminant health on various SWC approaches and the economic outcomes associated with their implementation at the farm level. The survey was disseminated through the Horizon Europe Thematic Network -SPARC, complemented by additional countries through the network of the COST Action ENVIRANT. Each participating country was represented by a designated national contact person. These national representatives were responsible for further distributing the survey within their respective livestock sectors via established local networks. Inclusion criteria required respondents to be active stakeholders within the ruminant livestock sector in the participating countries. Eligible participants included farmers, veterinarians, farm advisors, and researchers.

Participation in this survey was voluntary and anonymous, and all respondents were informed that there were no “right” or “wrong” answers and that their individual responses would not be evaluated. The following disclaimer was provided to all participants: “Participation in this survey is completely voluntary. All information you provide will be stored securely and will not be shared with anyone outside the research team. You will not be identifiable in any data collected from this survey”. The survey form consisted of 8 questions and three sections: (1) stakeholder information, capturing demographic and professional characteristics; (2) budget allocation, focusing on financial allocations directed toward worm control; and (3) an assessment of the perceived economic benefits associated with investing in seven selected SWC practices.

The study questions focused on the opinions of incurred costs in treating the animals and understanding of the various SWC practices. It is worth noting that participants were not asked to describe the specific strategies they use on their own farms; rather, they were asked to provide their general opinions about these topics.

To measure stakeholder perceptions, the survey utilized a 4-point scale (Strongly Disagree, Disagree, Agree and Strongly Agree). An additional ‘I don’t know’ category was also provided to ensure data accuracy and avoid forced responses. The survey was developed using Microsoft Forms (Microsoft 365) and translated into 11 languages (English, Dutch, French, Italian, Spanish, Polish, Greek, Latvian, Romanian, Lithuanian and Turkish) and distributed across 13 countries. To ensure conceptual and technical consistency across linguistic contexts, translations were performed by the National Contact Points (NCPs) within the SPARC and ENVIRANT networks. These individuals are senior researchers in veterinary parasitology, ensuring that the survey items were translated into culturally and professionally appropriate terminology for their respective livestock sectors.

The questionnaire was designed to be completed online within 3–6 min, although there was no time restriction imposed. To maximize stakeholder outreach, an additional dissemination campaign was carried out across the SPARC network using a wide range of channels, including SPARC National Contact Points, SPARC WhatsApp groups, social media, farmer events, and the SPARC EU webinar. A detailed table summarizing all survey items/questions and their corresponding response options is presented in the Supplementary File S1.

### 2.2. Data Analysis

During data cleaning, cases with incomplete responses or noticeable anomalies were addressed. Descriptive statistics were computed to characterize the study population and summarize stakeholder responses. Counts and percentages were calculated for categorical variables, including respondent role, age, gender, country of origin, and perceived worm control cost proportions. For the categorical variable ‘Country’, Belgium was used as the reference category based on alphabetical order. Due to the low participation numbers, UK responses were excluded from the analysis.

An ordinal logistic regression model was employed using the R package ‘ordinal’ (version 2023.12-4.1) to investigate factors associated with the perceived proportion of worm-control costs within respondents’ animal health budgets. The dependent variable consisted of four ordered categories (“<25%”, “25–50%”, “50–75%”, and “>75%”), reflecting increasing relative cost burden. Predictor variables included stakeholder roles, gender, age group, and country. Model coefficients were expressed as odds ratios (ORs) with corresponding 95% confidence intervals (CIs).

Perception of economic outcomes for seven SWC approaches was first reported visually for the two largest stakeholder groups—farmers (42.8%) and veterinarians (43.6%). The percentage of ‘Agreement’ reflects the combined frequency of ‘Agree’ and ‘Strongly Agree’ responses for each strategy. To assess overall receptiveness, a composite SWC Attitude Score was then derived by averaging the numeric responses for the seven individual practices (coded from 1 = ‘Strongly disagree’ to 4 = ‘Strongly agree’), where ‘I don’t know’ responses were excluded to ensure the score reflected active stakeholder opinions. Internal consistency of these seven approaches was evaluated using Cronbach’s alpha (α = 0.81). Associations between the SWC Attitude Score and respondent characteristics were examined using multiple linear regression with robust (HC3) standard errors. The same reference categories were used as the first regression model described above. Coefficients were reported as mean differences with 95% CIs.

All statistical analyses were conducted using R software (version 4.4.1; R Core Team, Vienna, Austria).

## 3. Results

### 3.1. Descriptive Statistics

A total of 1261 respondents completed the survey (Table 1). Participants represented a wide range of stakeholder groups, including farmers (42.8%), veterinarians (43.6%), researchers (7.1%), farm advisors (3.1%), and other professionals (3.4%) involved in livestock production and animal health. Respondents were distributed across 13 European countries, with the largest proportions from Türkiye (35.7%) and Italy (14.1%), followed by Belgium (9.8%), Latvia (8.3%), Greece (7.5%), and Romania (6.9%).

Most respondents were aged between 18 and 60 years. The largest age group was 18–30 years (30.8%), followed by 31–40 years (27.0%), 41–50 years (17.8%), and 51–60 years (14.9%).

**Figure 1:**
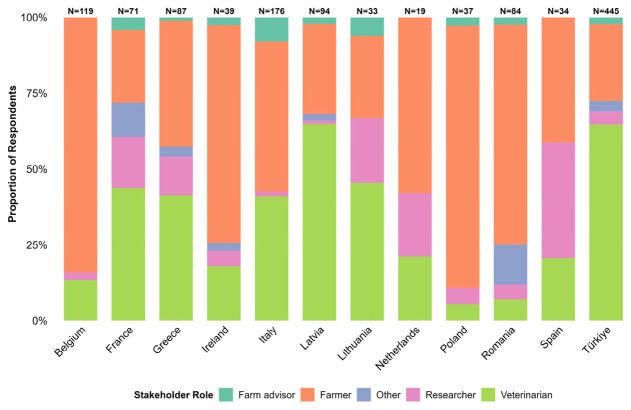
Distribution of survey respondent roles by country.

Stakeholder composition varied significantly across countries (Figure 1). Belgium, Poland, Romania, and Ireland were predominantly represented by farmers (cattle, sheep and goats combined) (>70%), whereas Türkiye and Latvia consisted mainly of veterinarians (>64%).

### 3.2. Perceived Proportion of Worm-Control Costs

**Figure 2:**
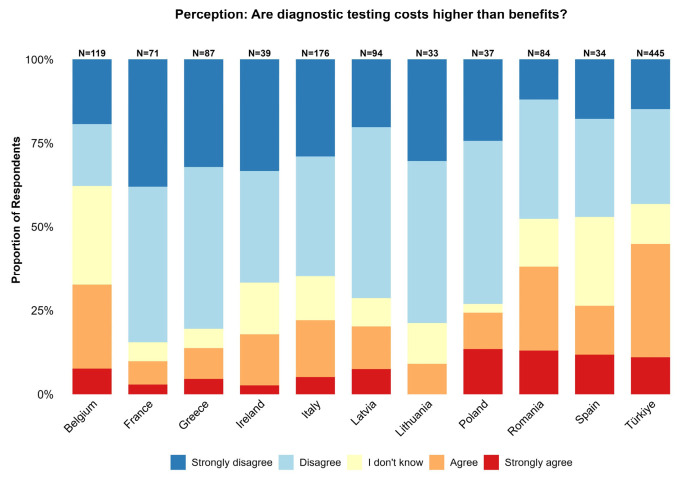
Participant agreement with the statement: “The costs of diagnostic testing for worm infections are higher than the benefits.” Disagreement (Strongly disagree/Disagree) indicates that benefits are perceived to justify the expense; agreement (Agree/Strongly agree) indicates that costs are perceived as excessive.

Overall, 56.7% of respondents disagreed with the statement that the costs of diagnostic testing are higher than the benefits, whereas 30.1% agreed and 13.2% selected ‘I don’t know’. It is worth noting that the disagreement % reflects perceived value rather than actual affordability or feasibility of diagnostics. However, perceptions varied considerably by country (Figure 2). Respondents in France, Greece, Lithuania, and the Netherlands were highly positive, with over 75% disagreeing that costs outweigh benefits. Conversely, Türkiye (44.9%) and Romania (38.1%) showed the highest proportions of respondents agreeing with the statement that costs exceed benefits. Belgium showed more divided opinions, with 37.8% disagreeing that costs outweigh benefits and 32.8% agreeing with the statement. Across all countries, “Strongly agree” was consistently the least frequent response.

**Figure 3:**
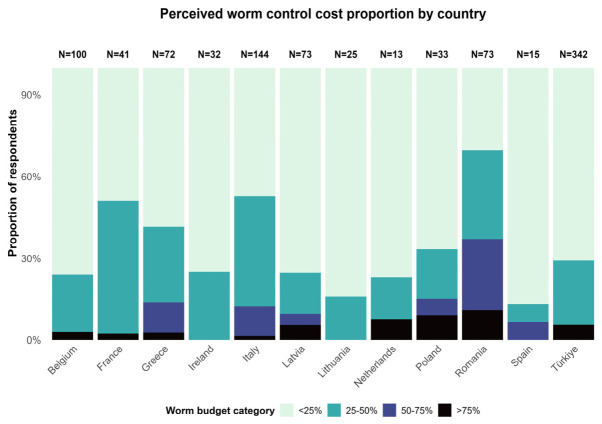
Perceived proportion of animal health budget spent on worm control costs across different countries (NA values were excluded).

Marked regional and stakeholder differences were observed in the perceived proportion of animal health budgets devoted to worm control (Figure 3). Across all regions, most respondents reported costs below 50%, although higher perceived cost burdens were reported in Romania.

Worm-control cost burden differed across respondent characteristics (Table 2). Compared with farmers, farm advisors were significantly less likely to report higher cost proportions (OR = 0.32, 95% CI: 0.10–0.85), while no significant differences with farmers were observed for veterinarians (OR = 0.90, 95% CI: 0.66–1.24) and researchers (OR = 0.57, 95% CI: 0.25–1.23). Male respondents reported lower cost proportions than females (OR = 0.70, 95% CI: 0.51–0.96). Marked country-level differences were observed, with respondents from Romania (OR = 9.58, 95% CI: 4.98–18.83), Italy (OR = 3.72, 95% CI: 2.09–6.77), France (OR = 3.16, 95% CI: 1.47–6.82), and Greece (OR = 2.63, 95% CI: 1.34–5.25) reporting significantly higher worm-control cost proportions compared with the baseline. No significant associations were found with age.

#### Perception of Economic Outcomes for Seven Major SWC Practices

**Figure 4:**
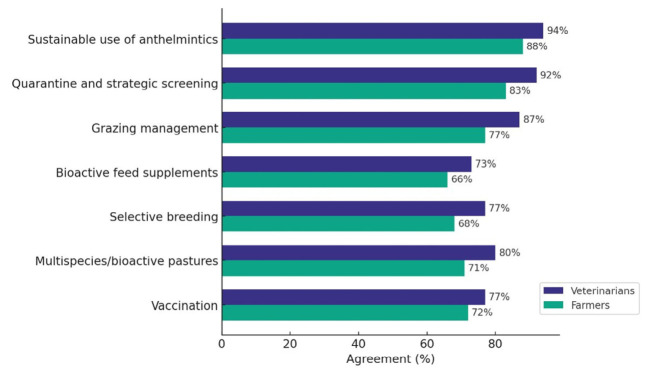
Summary of the farmers and veterinarians’ perception of the economic feasibility of different SWC approaches.

Figure 4 illustrates how the two key stakeholder groups (the largest stakeholder groups in the survey responsiveness), farmers (42.8%) and veterinarians (43.6%), perceived the economic feasibility of various SWC approaches. The most widely recognized method was the sustainable use of anthelmintics, with agreement from 94% of veterinarians and 88% of farmers. This was closely followed by quarantine and strategic screening (supported by 92% of the veterinarians and 83% of farmers) and grazing management (supported by 87% of veterinarians and 77% of farmers).

A majority of the respondents also agreed with other SWC approaches (bioactive feed supplements, selective breeding, multispecies/bioactive pastures and vaccination), although at somewhat lower percentages.

Responses to the seven SWC strategies were aggregated into an overall SWC Attitude Score, representing respondents’ mean level of agreement across practices. The index demonstrated good internal consistency (Cronbach’s α = 0.81). Based on the SWC Attitude Score, veterinarians had a slightly more positive attitudes towards SWC practices than farmers (β = 0.07, 95% CI: 0.002–0.13) (Table 3); male respondents lower than females (β = −0.11, 95% CI: −0.17 to −0.05); and respondents aged 51–60 years lower than those aged 18–30 years (β = −0.10, 95% CI: −0.19 to −0.01). Strong cross-country differences were observed, with respondents from Greece, Italy, Poland, Romania, Spain, and Türkiye reporting higher SWC attitude scores than those from Belgium (all *p* < 0.01). Overall, the model only explained a low proportion of variance in attitudes (Adjusted R^2^ = 0.06).

## 4. Discussion

This study sought to collect initial stakeholder perceptions regarding the economic feasibility of SWC approaches via a short survey across Europe. Gastrointestinal nematodes are widely recognized as a major constraint on ruminant health and productivity in Europe [23], although our needs-assessment results show that the level of awareness varies between countries. As indicated by reports from the SPARC project [32], farmers frequently encounter challenges in translating SWC approaches into practice. These challenges may often be attributable to constraints such as time, labor availability, limited diagnostic access, and the perceived complexity of SWC tools [32]. In addition, the existence of communication gaps between advisors and farmers, the fragmentation of knowledge-exchange pathways, farm-size, and the lack of clear, tailored, region and enterprise (farm sizes)-specific guidelines act as potential obstacles [9,33].

In our principal findings, the study showed substantial contrast between the theoretical appreciation of SWC approaches (Figure 4) and the perceived budget dynamics associated with diagnostics (Figure 2). Firstly, diagnostics were generally viewed as affordable (worth noting this was the perceived value rather than actual affordability). This could indicate that their perceived adoption is often motivated more by sustainability considerations than by direct economic motives. A similar study [34] reported that while anthelmintics are typically used for economic reasons, the decision to employ diagnostic tools is more closely associated with long-term sustainability goals, suggesting that cost is not the only primary factor determining diagnostic uptake [34]. This suggests that existing financial pressure may increase stakeholders’ desire for better control over outcomes. In this context, diagnostics could be perceived as a short-term expense rather than a long-term cost-saving measure. However, immediate labor and testing costs often discourage uptake in systems with tighter profit margins [35].

Secondly, the findings confirmed that roundworms remain a major animal health concern across Europe (Figure 3). This aligns with recent assessments showing that parasitic worm infections continue to impose sector-wide losses estimated at over €1.8 billion in Europe annually [9]. The observed differences between countries in the perceived importance of roundworms may relate to the differing prominence of the sheep sector, where GIN are consistently recognized as primary health challenges [9].

Thirdly, the observation that cost is not perceived as the main obstacle to SWC implementation suggests that other constraints influence farmers’ willingness or ability to adopt evidence-based parasite management strategies. Similar studies across Europe highlight potential obstacles such as a lack of time, complexity of messages, insufficient knowledge exchange, and limited/unclear practical guidance on sustainable approaches, all of which may hinder diagnostic uptake and broader SWC adoption [36]. Hence, future studies should focus on understanding and addressing these non-economic barriers, e.g., behavioral factors, perceived factors and practical feasibility of SWC approaches.

Beyond diagnostics, our results reveal a hierarchy of acceptance for other SWC approaches. The top three approaches in the hierarchy were targeted selective treatment, grazing management, and quarantine and strategic screening strategies, which were among the most positively viewed interventions. This could be because they can be applied immediately and do not rely on approaches that take a long time to implement (e.g., selective breeding), are not readily available (vaccines) or may have a variable efficacy (bioactive compounds). This is supported by previous studies suggesting that farmers are more likely to adopt strategies that integrate seamlessly into their existing workflows and management routines [35]. In contrast, the use of bioactive plants and nematode-trapping fungi received the highest uncertainty answers (“I don’t know”), particularly in Western European countries. Although these methods show promise in controlled trials [15,37], the observed stakeholder hesitation may reflect a lack of knowledge of these control methods, concerns regarding their variable efficacy under field conditions, and/or the limited availability of commercial products in these regions. Nonetheless, also for these more variable approaches, the overall perception was positive (>60%), indicating that they also have a good place in the European basket of SWC options. While these models reveal significant associations, the low Adjusted R^2^ (0.06) indicates that demographic factors explain only a small portion of the variance in perceptions. This suggests that adoption is likely driven by more complex, unmeasured factors such as an individual farm history, local peer networks, or specific psychological drivers.

Stakeholder factors showed a consistent trend observed across all regions, with a divergence in attitudes between professional advisors and farmers. Veterinarians displayed significantly higher acceptance of knowledge-intensive practices than farmers, although the difference was small (8%). A higher positive attitude of veterinarians may be logical, as SWC will require more in-depth knowledge on parasite epidemiology and control than traditional control based on the regular administration of anthelmintics on fixed calendar days. Therefore, SWC approaches may need more specialist advice and put the veterinarians in a key position to drive their adoption [23,35]. There was a disconnect with farm advisors (n = 40) who were over three times less likely than farmers to report that worm control accounts for a high proportion of their animal health budget. This observed discordance between farmers and advisors is consistent with earlier findings [38], who reported that the adoption of targeted (selective) treatments is influenced not only by economic considerations but mainly by practical challenges that vary across stakeholders.

Demographic factors also played a subtle but significant role in shaping perceptions. Male respondents reported both lower cost proportions and lower overall SWC Attitude Scores compared to females. Furthermore, older respondents (51–60 years) held fewer positive attitudes towards SWC than those aged 18–30. The gender and age-related differences in farmer attitudes and behaviors have been previously identified in the context of sustainable parasite control adoption [35]. These variations highlight the complexity of stakeholder attitudes, suggesting that sociological drivers play a key role and warrant specific investigation into future sustainable farming research.

Future field studies could validate these perception-based findings by implementing and testing different SWCs while accessing farm performance data [25]. Simultaneously, other challenges that may act as important obstacles to the uptake of SWC should be further investigated. These may include challenges related to low awareness, practical feasibility, unclear or conflicting operational guidance and contradictory advice by different farm advisors [5].

The primary limitation of this study was the uneven geographical distribution of respondents and the variation in stakeholder composition across surveyed countries. Notably, a significant proportion of the responses were from Türkiye, which is attributable to the substantial size of its livestock sector and the active engagement of its veterinary networks. While this provides valuable insight into practices and perceptions within a major European production context, this geographical concentration means that overall European-level results may be disproportionately influenced by Turkish responses. Furthermore, differences in the relative proportions of farmers and veterinarians across different countries may have had an impact on the perspectives captured, consequently limiting the comparability of cross-country results. Therefore, the observed country-level variations must be interpreted with caution due to the highly unequal sample sizes and the confounding effects of national agricultural policies and sector-specific production systems. It is also worth noting that harmonized population data for livestock stakeholders across the 13 participating countries are unavailable, and statistical weighting was not applied. Hence, these findings should be interpreted as an exploratory snapshot of this specific cohort rather than a representative European census. Moving forward, the study can be extended to other European countries, including the Scandinavian region.

Practical limitations were that participation still required consistent internet access and a minimum level of digital literacy, which may have excluded individuals lacking these resources. Populations residing in areas with limited connectivity are also likely to be underrepresented. The study is also subject to self-selection bias, as respondents who chose to participate voluntarily may differ systematically from those who did not, particularly with respect to interest, motivation, and the strength of their views. It is recommended that future studies implement a more harmonized and balanced sampling design, with the objective of enabling robust in-depth cross-country comparisons.

These results suggest that the overall hurdles to adoption are most effectively addressed via policy-supported, bottom-up approaches where farmers, veterinarians, and advisors co-develop solutions. Specifically, for veterinarians, this implies a shift toward a consultative role focused on specialized parasite management guidance. For policymakers, the focus should prioritize the funding and development of national and regional Communities of Practice (CoP) [39]. A central recommendation is the strategic use of SPARC ambassadors—key opinion leaders who facilitate horizontal knowledge transfer. By leveraging these networks to disseminate locally adapted materials, policymakers and industry leaders can bridge the gap between scientific efficacy and on-farm reality.

## 5. Conclusions

Our study’s findings, drawn from a voluntary participation survey, indicate that a significant proportion of stakeholders across the surveyed European countries perceive SWC approaches to be economically feasible. This observation suggests that cost is unlikely to be the only primary obstacle to adoption. Diagnostics are also generally viewed as beneficial, suggesting a substantial scope for increasing their uptake. However, it is important to note that the uneven geographical distribution of respondents and the variation in stakeholder composition across surveyed countries limit the generalisability of the results. The present findings demonstrate an exploratory snapshot of a specific cohort within European countries, thus providing insights into the assessed topic, as opposed to insights from the entire European region.

The study revealed significant variations in perception across the different surveyed countries. These findings highlight the need for adapted, region-specific SWC approaches, rather than the use of uniform recommendations. In the surveyed countries, field-based evaluations are essential to confirm these perceptions under real-world conditions. Simultaneously, non-economic barriers, including practical implementation challenges and communication hurdles, must be addressed through collaborative structures exemplified by the SPARC Communities of Practice. These initiatives aim to support sustainable, locally grounded, farmer-led, and veterinarian-supported worm control strategies.

## Figures and Tables

**Table 1:** General characteristics of survey respondents (n = 1261). The Other category includes technicians, policy makers and industry suppliers.

Variable	Category	Count	Percent
**Age**	18–30 years	389	30.8
	31–40 years	341	27.0
	41–50 years	224	17.8
	51–60 years	188	14.9
	>60 years	116	9.2
	<18 years	3	0.2
**Role**	Veterinarian	551	43.7
	Farmer	540	42.8
	Researcher	90	7.1
	Farm advisor	40	3.2
	Other	40	3.2
**Country**	Türkiye	445	35.7
	Italy	176	14.1
	Belgium	119	9.5
	Latvia	94	7.5
	Greece	87	7.0
	Romania	84	6.7
	France	71	5.7
	Ireland	39	3.1
	Poland	37	3.0
	Spain	34	2.7
	Lithuania	33	2.6
	Netherlands	19	1.5
	United Kingdom	6	0.5
**Gender**	Male	849	67.3
	Female	400	31.7
	Prefer not to say	12	1.0

**Table 2:** Ordinal logistic regression of factors associated with perceived worm-control cost proportion. Note: Significance levels: * *p* < 0.05, ** *p* < 0.01, *** *p* < 0.001.

Predictor	OR	Std. Error	z Value	*p* Value	2.5% CI	97.5% CI	Significance
**Role (ref: Farmer)**							
Farm advisor	0.320	0.531	−2.145	0.032	0.102	0.846	*
Researcher	0.571	0.403	−1.393	0.164	0.250	1.226	
Veterinarian	0.901	0.162	−0.645	0.519	0.655	1.238	
**Gender (ref: Female)**							
Male	0.697	0.164	−2.199	0.028	0.506	0.963	*
Prefer not to say	0.431	0.839	−1.005	0.315	0.061	1.916	
**Country (ref: Belgium)**							
France	3.158	0.391	2.943	0.003	1.468	6.819	**
Greece	2.634	0.348	2.782	0.005	1.336	5.251	**
Ireland	1.032	0.470	0.067	0.947	0.392	2.518	
Italy	3.717	0.299	4.391	<0.001	2.090	6.770	***
Latvia	1.027	0.379	0.072	0.943	0.485	2.152	
Lithuania	0.619	0.609	−0.786	0.432	0.164	1.889	
Netherlands	0.940	0.704	−0.088	0.930	0.197	3.408	
Poland	1.575	0.458	0.992	0.321	0.629	3.826	
Romania	9.579	0.339	6.666	<0.001	4.975	18.833	***
Spain	0.588	0.804	−0.660	0.509	0.087	2.386	
Türkiye	1.514	0.309	1.344	0.179	0.835	2.810	
**Age (ref: 18–30 years)**							
31–40 years	0.851	0.188	−0.857	0.391	0.588	1.228	
41–50 years	0.855	0.220	−0.712	0.477	0.554	1.313	
51–60 years	0.984	0.231	−0.070	0.944	0.624	1.545	
>60 years	1.028	0.289	0.094	0.925	0.579	1.805	

**Table 3:** Associations between respondent characteristics and the SWC Attitude Score. Note: Significance levels: * *p* < 0.05, ** *p* < 0.01, *** *p* < 0.001.

Predictor	Estimate	Std. Error	t Value	*p* Value	2.5% CI	97.5% CI	Significance
**Role (ref: Farmer)**							
Farm advisor	−0.086	0.102	−0.843	0.399	−0.287	0.114	
Other	−0.035	0.077	−0.454	0.650	−0.187	0.116	
Researcher	0.002	0.062	0.032	0.975	−0.119	0.123	
Veterinarian	0.068	0.034	2.013	0.044	0.002	0.133	*
**Gender (ref: Female)**							
Male	−0.105	0.031	−3.449	0.001	−0.165	−0.045	***
Prefer not to say	−0.342	0.149	−2.298	0.022	−0.633	−0.050	*
**Country (ref: Belgium)**							
France	0.170	0.071	2.375	0.018	0.029	0.310	*
Greece	0.366	0.067	5.438	<0.001	0.234	0.498	***
Ireland	0.255	0.081	3.144	0.002	0.096	0.414	**
Italy	0.277	0.061	4.575	<0.001	0.158	0.396	***
Latvia	0.154	0.069	2.225	0.026	0.018	0.289	*
Lithuania	0.228	0.093	2.461	0.014	0.046	0.410	*
Netherlands	0.346	0.103	3.363	<0.001	0.144	0.547	***
Poland	0.370	0.085	4.344	<0.001	0.203	0.537	***
Romania	0.417	0.071	5.895	<0.001	0.278	0.555	***
Spain	0.467	0.096	4.890	<0.001	0.280	0.655	***
Türkiye	0.251	0.062	4.066	<0.001	0.130	0.372	***
**Age (ref: 18–30 years)**							
31–40 years	−0.025	0.036	−0.687	0.492	−0.096	0.046	
41–50 years	0.027	0.044	0.621	0.535	−0.059	0.114	
51–60 years	−0.103	0.045	−2.258	0.024	−0.192	−0.013	*
>60 years	0.073	0.053	1.385	0.166	−0.031	0.177	

## Data Availability

The data presented in this study are available on request from the corresponding author.

## Supplementary Materials

The following supporting information can be downloaded at: https://www.mdpi.com/article/10.3390/ani16101552/s1, File S1: Stakeholders survey questionnaire.

## Abbreviations

The following abbreviations are used in this manuscript:

SWC	Sustainable Worm Control
SPARC	Sustainable Parasite Control
GIN	Gastrointestinal Nematode
AR	Anthelmintic Resistance
FEC	Fecal Egg Count
TST	Target Selective Treatment
TT	Targeted Treatment
