# Supplementary material for: Perceptions on the Economic Feasibility of Sustainable Roundworm Control Practices in Grazed Livestock—A Short Survey Among European Farmers and Veterinarians
Source: Animals (Basel). 2026 May 19;16(10):1552. doi: 10.3390/ani16101552 (PMC13203688; doi:10.3390/ani16101552)
Supplement: Supplementary file 1 [file animals-16-01552-s001.zip › Supplementary File S1.pdf]

SUSTAINABLE WORM CONTROL IN GRAZING RUMINANTS

STAKEHOLDERS SURVEY QUESTIONS

(The survey was eventually translated into 11 local European languages from the original language that was English). The survey was conducted in February to June 2025.

Q1. Which of this best describes you?

- Cattle Farmer
- Sheep Farmer
- Goat Farmer
- Cattle & Sheep/Goat Farmer
- Veterinarian
- Consultant
- Researcher
- Others (please specify)

Q2. Which age group best describes you?

- <18 years
- 18-30 years
- 31-40 years
- 41-50 years
- 51-60 years
- >60 years

Q3. How do you identify your gender?

- Male
- Female
- Other

- Prefer not to say

Q4. In which country is your farm/company/organization/institution located?

- Albania
- Belgium
- Cyprus
- France
- Germany
- Greece
- Hungary
- Italy
- Ireland
- Latvia
- Lithuania
- Netherlands
- Poland
- Romania
- Spain
- United Kingdom
- Other (please specify)

Q5. What proportion do costs related to worm control take out of your animal health budget?

- <25% (less than)
- 25-50%
- 50-75%
- >75% (more than)
- Not applicable

Q6. I feel the costs/expenses of diagnostic testing for worm infections are higher than the benefits.

Strongly disagree	Disagree	Agree	Strongly agree	I don't know

Q7. I feel that Sustainable Worm Control practices can improve the profitability in farms (e.g. reduced losses, increased performance, reduced medical costs, improved animal health).

Strongly disagree	Disagree	Agree	Strongly agree	I don't know

Q8. Do you think the following methods could be an economically beneficial way to control worm infections on the farm?

**Methods**	**Strongly disagree**	**Disagree**	**Agree**	**Strongly agree**	**No opinion/ I don't know**
1. Sustainable use of anthelmintics (e.g. Targeted treatment, Targeted Selective Treatment).					
2. Quarantine and strategic parasite screening for new livestock arrivals.					
3. Grazing management (e.g. rotational grazing, mixed grazing with different animal species, pasture mowing).					
4. Feed supplements based on bioactive plants with anthelmintic properties					

5. Selective breeding for making animals less susceptible to worms.					
6. Use of pastures with multispecies swards or seeded with bioactive plants					
7. Vaccination.					
